# Towards Network Lifetime Enhancement of Resource Constrained IoT Devices in Heterogeneous Wireless Sensor Networks

**DOI:** 10.3390/s20154156

**Published:** 2020-07-26

**Authors:** Muhammad Salah ud din, Muhammad Atif Ur Rehman, Rehmat Ullah, Chan-Won Park, Byung Seo Kim

**Affiliations:** 1 Department of Electronics & Computer Engineering, Hongik University, Sejong City 30016, Korea; salah_udin@outlook.com (M.S.u.d.); atif_r@outlook.com (M.A.U.R.); 2 Department of Computer Engineering, College of IT Convergence, Gachon University, Seongnam 13120, Korea; rehmatullah@gachon.ac.kr; 3 Electronics and Telecommunications Research Institute, Daejeon 34129, Korea; cwp@etri.re.kr; 4 Department of Software and Communications Engineering, Hongik University, Sejong City 30016, Korea

**Keywords:** clustering, energy consumption, MAC, TOPSIS, WSNs, internet of things, network lifetime, load-balancing

## Abstract

The participating nodes in Wireless Sensor Networks (WSNs) are usually resource-constrained in terms of energy consumption, storage capacity, computational capability, and communication range. Energy is one of the major constraints which requires an efficient mechanism that takes into account the energy consumption of nodes to prolong the network lifetime. Particularly in the large scale heterogeneous WSNs, this challenge becomes more critical due to high data collection rate and increased number of transmissions. To this end, clustering is one of the most popular mechanisms which is being used to minimize the energy consumption of nodes and prolong the lifetime of the network. In this paper, therefore, we propose a robust clustering mechanism for energy optimization in heterogeneous WSNs. In the proposed scheme, nodes declare themselves as cluster head (CH) based on available resources such as residual energy, available storage and computational capability. The proposed scheme employs the multi criteria decision making technique named as Technique for Order of Preference by Similarity to Ideal Solution (TOPSIS) which allows the child nodes to select the optimal CH among several potential CH candidates. Moreover, we also propose mechanisms such as CH-acquaintanceship and CH-friendship in order to prolong the network lifetime. Simulation results show that our proposed scheme minimizes the control overhead, reduces the power consumption and enhances overall lifetime of the network by comparing with the most recent and relevant proposed protocol for WSNs.

## 1. Introduction

Wireless Sensor Networks (WSNs) have wide range of applications such as environmental monitoring [1], habitat monitoring [2], military applications [3], weather monitoring [4], smart grid [5], traffic monitoring [6] and forest fire detection [7] etc. In WSNs, plethora of small-sized application-specific sensor nodes are randomly deployed in the sensor field. These sensor nodes collect information and forward it towards their managing node (sink node or cluster head (CH)) via wireless communication. The nodes are resource constrained in terms of energy, computational capability, storage capacity, and communication range. The WSN nodes are battery operated and replacement of battery might not be possible due to harsh terrains and severe environmental conditions. When the nodes deplete their whole energy, the network may not operate properly and may result in performance degradation. Therefore, an efficient energy management scheme is of paramount importance to minimize the energy consumption of sensor nodes in order to prolong the lifetime of network.

**Figure 1:**
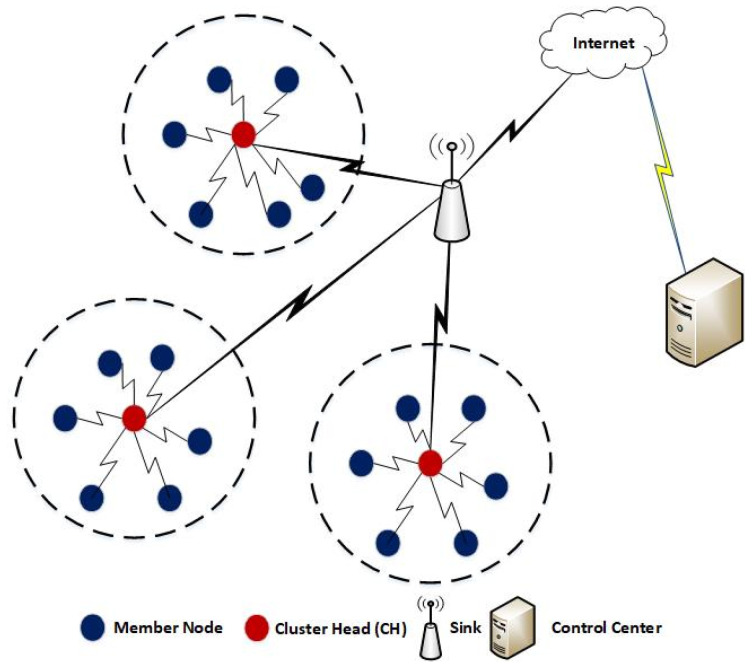
Clustering in Wireless Sensor Networks (WSNs).

To minimize the energy consumption and to prolong the lifetime of nodes in WSNs, several efforts have been devoted in the literature such as MAC protocols [8], routing protocols [9], and clustering algorithms [10,11], etc. Clustering algorithms are the most prominent mechanisms to make WSNs energy efficient. These algorithms organize a WSN in multiple clusters of various sizes as shown in Figure 1. A single node among each cluster is elected as CH and the member nodes can communicate directly with their respective CH located at the shorter distance which in turn reduces the transmission energy of node as compared to direct or multi-hop transmissions towards the sink node.

Clustering algorithms can be classified based on: (1) the parameters considered for the CH election (a detailed description of CH election approaches is presented in Section 2) and (2) the nature of the execution of the clustering algorithm (e.g., centralized or distributed) [12]. Considering the nature of execution, these algorithms can be either centralized (e.g., sink node decides the CHs in the network) or distributed (e.g., the nodes execute the CH election process locally). The centralized algorithms require the complete topology information. Therefore, the efficiency of centralized algorithms may degrade in large scale WSNs, as collecting the entire network information at the sink node or base station may result in high energy consumption and brings on large delays. On the other hand, distributed algorithms are highly efficient for large scale WSNs. In distributed algorithms, each node decides its role (e.g., to declare itself as a CH or to associate with CH) based on the information of its neighboring nodes [11,12].

Clustering in WSNs is very beneficial to prolonging the network lifetime and reducing the energy consumption of the nodes. It is shown that clustering improves the network lifetime by 2 to 3 times more than that of non-clustering schemes [13]. Without clustering, significant amount of energy is consumed in data transmission, where nodes communicate with the sink node directly which may result in excessive amount of energy consumption. However, in clustering, nodes only communicate with the CH located at a shorter distance rather than the sink node which may be located far away from the node. Therefore, an efficient organization of sensor nodes into clusters is of extreme importance for reducing overall energy consumption in the network.

Most of the existing cluster-based schemes are developed for homogeneous networks [14]. These schemes only consider homogeneous nodes in the network e.g., those nodes have identical resources such as residual energy, computational capability, and storage capacity. Therefore, it is highly likely that each node in the network may have an equal probability of becoming CH. In the equi-probable CH selection mechanism, there is a possibility of electing CH having low residual energy as compared to the one with a comparatively higher energy level. However, we are considering heterogeneous WSNs [15], where the resources of nodes may vary in terms of computational capability, residual energy, and storage capacity. In most of the existing works in heterogeneous WSNs, CH is selected based on residual energy, distance from member nodes, and distance from the sink node. However, to the best of our knowledge, all such schemes do not consider the traffic load, computational capability, and remaining storage of the CH. In the heterogeneous environment, the traffic generated by nodes may not be identical, meaning that traffic could be scalar (e.g., temperature, humidity, pressure etc.) and/or multimedia (e.g., audio, video). Therefore, the traffic load on the CHs may also vary. For instance, a CH that manages multimedia traffic would be overloaded due to high computations, and more storage requirements as compared to CH managing scalar traffic. As a result, energy consumption may also vary according to the traffic class on each CH.

To address the aforementioned challenges, in this paper, therefore, we propose an energy-efficient clustering scheme for heterogeneous WSNs. The main contributions of the proposed scheme can be summarized as follows:

- We propose an efficient CH declaration scheme to reduce the energy consumption of nodes and to prolong the network lifetime. The propose scheme provides a mechanism through which a node declares itself as a CH based on the available resources such as residual energy, computational capability, and available storage. Once the CH is declared, it remains CH until the resources fall short than a certain threshold level.
- For the un-associated nodes, we employ the multi-criteria decision-making technique known as Technique for Order of Preference by Similarity to Ideal Solution (TOPSIS) to select an optimal CH.
- We also provide mechanisms of CH-acquaintanceship and CH-friendship to reduce the energy consumption, optimize the workload, minimize the packet drop rate, and extend the lifetime of CH. In acquaintanceship mechanism, the CHs in the network may collaborate with each other for mutual benefits. Whereas, in CH-friendship, the low resources CH may request high resources CH to perform operation on behalf of low resources CH to avoid early failure and data loss.
- Simulations are performed in Castalia (OMNET++) to reveal the performance of the proposed scheme with relevant and state of art scheme in terms of CH lifetime, re-clustering frequency, packets loss, control overhead and average energy consumption of the network.

The rest of the paper is organized as follows: Section 2 is devoted to background and related work. Section 3 describes the problem scenario. In Section 4, we present our proposed scheme. Performance evaluations are presented in Section 5 and finally, Section 6 draws the conclusion.

## 2. Background and Related Work

### 2.1. Clustering Overview

In this section, we discuss the basic operations of the clustering mechanism in WSNs as follows [11].

1. The whole network is partitioned into clusters.
2. After cluster formation process, the selected CHs gather and aggregate the data received from member nodes and transmit it towards the sink node. Usually, the CHs consume more energy as compared to the other nodes and ran out of power due to high load. Therefore, to balance the energy consumption, the role of CH is switched among different sensor nodes, meaning that a CH may not be CH for longer time in the network and other high resources sensor nodes can take over the role of CH. However, in order to elect an optimal CH, the following strategies may be adopted and are discussed as follows:
  - Deterministic CH election: In deterministic schemes, CHs are super-nodes having high resources such as energy, storage, and computational capability etc.
  - Random CH election: In these schemes a CH is elected based on randomly generated value.
  - Adaptive CH election: Instead of electing CH randomly, adaptive CH election schemes provide a mechanism to elect CH based on several parameters such as residual energy, computational capability, storage, distance, etc. The combination of the multiple parameters is utilized to elect an optimal CH among several potential candidates.
3. After CHs election, each CH broadcasts its information of becoming a CH to other nodes in its communication range. The receiving nodes may receive information from several CHs in its vicinity and decide which CH to join based on several metrics such as distance from CH, computational capability, residual energy and storage capability of CH, etc. After joining a particular CH, the node forwards its sensed data towards that CH.
4. When resources of a current CH falls below a certain threshold, re-clustering is performed to avoid the data loss. However, frequent re-clustering degrades the performance of network due to control overhead.

### 2.2. Related Work

A myriad of energy efficient schemes have been proposed for WSNs. The one with the most hype around is low energy adaptive clustering hierarchy (LEACH) [16]. LEACH is one of the pioneer routing protocols proposed for energy optimization in WSNs. The basic idea of LEACH is to balance the energy consumption by rotating the CH role among the nodes. Each node generates a random number between 0 and 1. If the random number is less than a certain threshold, the node declares itself as a CH. In LEACH, each node in the network has an equal probability to be elected as CH irrespective of its residual energy. In this equal probability CH selection mechanism, there is a possibility of electing CH having lower residual energy compared to the one with a higher energy level. LEACH is then enhanced to R-LEACH in [17], which provides a CH selection mechanism based on the node’s initial energy, residual energy and the optimal number of CHs in the network. In [18], low energy adaptive clustering hierarchy centralized (LEACH-C) mechanism was proposed. LEACH-C modifies the existing LEACH protocol. In LEACH-C, each node in the network forwards location and residual energy information to the sink node. Based on the received information, the sink node performs clustering and determines the optimal CH for each cluster. Due to the centralized nature of LEACH-C, communication overhead increases in the re-clustering process.

In [19], a sleep-wake energy-efficient distributed clustering algorithm (SEED) for wireless sensor networks was proposed. SEED divides the sensing network into three regions based on energy level (e.g., low energy region, advance energy region, high energy region) to achieve even energy distribution. In SEED, nodes which have high residual energy in each region can only become CHs. However, SEED results in additional control messages overhead that highly effects the lifetime of the network. Moreover, SEED does not take into account the node(s) storage capacity, and computational capability during CH declaration process which may degrade the overall performance of network in high multimedia traffic conditions.

In [20], an Energy Centric Cluster-Based Routing Protocol (ECCR) for WSNs was proposed. In ECCR, clusters are predetermined and static, and CHs are selected based on the node’s rank where rank comprises of node’s residual energy and average distance from member nodes. The nodes with high residual energy and low average distance have a high rank. ECCR also resulted in control messages overhead which increases the power consumption of the nodes.

A dual and static CH selection mechanism was proposed in [21] that split the entire network into equal sized static clusters. In this scheme, two CHs are selected in each cluster e.g., one for data aggregation and another for data transmission. The CHs are selected based on residual energy and distance from child nodes and sink node. The CH which has low distance from member nodes is used for data aggregation while the other CH which has low distance from sink node is responsible for data transmission. The selection of two CHs generates additional control overhead in the network. Furthermore, this scheme only considers residual energy and distance for CH selection which may result in inefficient CH selection in a heterogeneous network environment.

An energy-driven unequal clustering protocol (EDUC) [22] was proposed to minimize the energy consumption in re-clustering (i.e., CH node rotation). In EDUC, a node can act as a CH only once during the entire network lifetime. Another scheme named as energy and proximity based unequal clustering algorithm (EPUC) [18] was proposed to address the energy imbalance issue of the nodes closer to the base station (BS) due to excessive relaying of data. In this scheme, nodes are selected as CHs based on their residual energy and BS proximity. In [23], an extension of LEACH protocol named as stable election protocol (SEP) was proposed. SEP ensure the uniform energy consumption of all the nodes in the network to prevent the early depletion of nodes.

In [24], an energy efficient multi level and distance aware clustering (EEMDC) was proposed. EEMDC splits the entire network into three levels based the number of hops from the base station. These levels comprised of first level clusters which have hop counts of one to two, the second level clusters having hop counts of three to five and third level clusters which have hop counts of six or more. The different levels determine the distance from base station (e.g., the nodes in level 1 are closer to BS than that of nodes in level 3). In EEMDC, CH declaration is based on residual energy and hop-count. EEMDC ensures the smallest route towards the BS in order to optimize the energy consumption and enhance the overall network lifetime.

An energy-aware routing algorithm (ERA) has been proposed in [25]. In ERA, the CH declaration mechanism is based on residual energy of nodes. Meaning that, the nodes which have high residual energy are elected as CH. ERA generates directed virtual backbone (DVB) of CHs to relay the data from CHs towards the sink. Sink initiate process of DVB generation by broadcasting a route request message towards the CHs. On recieving route request message, the CH(s) increment its level by one, higher than that of sink (e.g., consider the level (L) of the sink node is zero e.g., L(sink) = 0, then L(CH) = L(sink) + 1) and then re-broadcast the message to other CH nodes in their vicinity and creates a DVB. A CH selects the relay nodes based on the ratios of the average residual energy of the CHs in different levels and distributes all the aggregated data packets towards the sink node sequentially.

Energy and coverage aware distributed clustering (ECDC) has been proposed in [26]. In ECDC, the node declare itself as a CH based on its residual energy and coverage. Each node share its residual energy and coverage information with its neighboring nodes. The node which has high residual energy and coverage is selected as a CH. ECDC achieves a lower energy consumption of nodes and better in-coverage performance compared to other protocols.

A decentralized energy-efficient hierarchical cluster-based routing algorithm (DHCRA) was proposed in [27]. In DHCRA, CHs are selected at tree edges based on residual energy and distance from BS. DHCRA minimizes control overhead and optimizes the energy consumption of nodes. In [28], an adaptive clustering algorithm (TCAC) has been proposed to enhance the lifetime of the network. TCAC enables the CHs to adjust their power level to achieve the desired network connectivity. TCAC generate the balanced clusters across the whole network and improves the network lifetime. However, in TCAC, the periodic transmission of range updates and competition-based CH selection increases the network complexity.

Link-aware clustering mechanism (LCM) [29] for WSNs has been proposed. In LCM, the CHs are elected based on the condition of the links and status of sensor nodes. In LCM, predicted transmission count (PTX) has been introduced to evaluate the condition of the node(s). PTX is calculated based on the residual energy, transmission power, and the link quality with a specific neighbor. The node which has a large PTX value has significant chance to become a CH.

Cluster chain weighted metrics (CCWM) has been proposed in [30] to optimize the energy consumption and enhance the performance of WSNs based on weighted metrics. In CCWM, the CHs are elected based on weighted metrics. In this scheme, the child nodes forward the sensed data directly towards the CH whereas the CH transfer the aggregated data towards the neighbor CH until the data reach the BS. CCWM improves the network lifetime however, the direct intra-cluster communication may leads to unfair energy distribution. A non-probabilistic multi-criteria based CH selection mechanism was proposed in [31]. In this scheme, analytical network process decision tool is used for CH is selection. A collection of various parameters have been collected and the best parameters among the collected ones are selected for CH selection.

**How does the proposed scheme differ from prior works?** In most of the existing cluster-based schemes in WSNs, CH declaration is based on residual energy, distance from member nodes, and distance from the sink node. However, to the best of our knowledge, all such schemes do not consider storage capacity and computational capability for best CH declaration. Besides, most of the existing proposals do not take into account one or more of the following CH and sensor node parameters in new node association process: (i) traffic load on CH; (ii) residual energy; (iii) computational capability of a CH; (iv) distance between CH and sink node; and (v) less distance between a sensor node and a CH. In contrast to the existing schemes, our scheme proposes an efficient mechanism for the new node association, and considers all of the aforementioned parameters for optimal CH selection. Furthermore, the proposed scheme also provides CH-acquaintanceship and CH-friendship mechanism in order to prolong the network lifetime.

## 3. Problem Scenario

Before our proposed scheme is discussed, it is important to describe the problem scenario. After that the proposed scheme is presented.

Most WSN applications generate bursty traffic and the nodes in such applications may not be homogeneous (e.g., multimedia nodes, scalar nodes). For instance, the habitat monitoring and more particularly the wildlife monitoring is one of the realistic applications where nodes may be heterogeneous and high capacity nodes (multimedia/camera) may be deployed close to low capacity nodes (e.g., scalar). The WSN based wildlife monitoring systems have gained significant importance for monitoring and protection of wildlife species. These applications are beneficial in animal detection, animal behavior detection, and environmental observation. In wildlife monitoring systems, several heterogeneous sensor nodes (e.g., scalar and multimedia) are widely deployed for habitat environment observation, such as temperature, humidity, wind, animal movements, etc. [32,33]. These sensor nodes work collaboratively to monitor the sensor field. In order to analyze the condition of animals (e.g., animals health, food and water conditions, behavioral condition, etc.) in severe environmental conditions, the low resources scalar nodes sense the temperature, humidity, wind conditions while the camera nodes capture the visual information and forward the information towards the base station(s). When the scalar nodes detect the variation in environmental factors, these nodes may inform the available camera nodes in their vicinity to capture the effect and finally transfer the captured data towards the sink or base station. The concerned authorities (e.g., wildlife department) may utilize the gathered data to analyze the effect of environmental condition on wild species and may take necessary measures accordingly.

These heterogeneous nodes generate various kind of traffic such as audio, video, and scalar as discussed before. In case of event detection, multiple nodes in the vicinity that detect the event may generate a massive amount of data simultaneously and transmit the data toward the CHs. Due to bursty traffic, the CHs may be overloaded as they receive and manage data from all the members of cluster. This may affect the lifetime of CHs. Therefore, minimizing the energy consumption of CHs plays a vital role to prolong the lifetime of the WSNs. In order to enhance the network lifetime, an efficient CH declaration mechanism is of upmost importance.

In the literature, several approaches have been devoted for efficient CH declaration [16,17,18,19]. These proposed schemes consider residual energy, distance from member nodes, and distance from the sink node as important parameters for a node to be selected as a CH. However, these approaches fall short for the case of high-volume traffic. If the selected CH has high residual energy and short distance from the sink node and member nodes, however, the storage and computational capability (capability of a node to execute instructions per unit time) is low, the CH may not accommodate the incoming data received from cluster members effectively. This may result in packet drop and re-transmissions of packets in the network. Similarly, due to low computational capability, the CH may spend more time in data processing, thereby, consumes more energy. Furthermore, the frequency of re-clustering increases which ultimately brings extra control overhead in the network. Therefore, the storage and computational capability of a node are the most stringent factors for optimal CH and to mitigate packet drops and re-transmissions in the network.

## 4. Proposed Scheme

We propose an efficient clustering scheme that comprises of various phases such as CH declaration phase, node association phase, CH-acquaintanceship phase, and CH-friendship phase. In the proposed scheme, we consider a scenario where heterogeneous wireless sensor nodes are randomly deployed in a network. The deployed nodes are further divided into groups named as clusters. Each cluster has one CH node responsible for data collection from its cluster members, data aggregation and data transmission towards the sink node. The nodes declare themselves as CH based on available resources such as residual energy, storage and computational capability.Once the CHs declaration process completes and the CHs are declared, the nodes association process executes in which nodes select the best CH among several potential CH candidates by employing the multi-criteria decision-making technique known as TOPSIS [34]. The proposed scheme also presents a robust mechanism of CH-acquaintanceship and CH-friendship in order to balance the load on the CH and to prolong the lifetime of the CH. In the following subsections, we present all the aforementioned phases in detail.

### 4.1. Cluster Head (CH) Declaration Phase

In the heterogeneous WSNs, the available resources of nodes may not be identical, meaning that the nodes may be different in terms of storage, residual energy and computational capability. Therefore, when a node participates in CH declaration process, it is very important to consider the available resources and those resources must be higher than a certain threshold value. For instance, if a node has residual energy greater than minimum residual energy $\left(E_{min}\right)$, computational capability greater than minimum computational capability $\left(C_{min}\right)$ and storage capacity greater than minimum storage $\left(S_{min}\right)$, the node is eligible to become a CH. Moreover, as the values of resources are measured in different scales, these values need to be normalized to a common scale. To this end, we employ a min-max normalization technique to normalize the available resources values between a specific range (e.g., [*x* *y*]) and is defined as follows.

(1) $$R_{i}\;=\;\;x+\frac{\left(r_{i}-\;\;\min\left(r_{i}\right)\right)\;\;\times \;\;\left(y-x\right)}{max\left(r_{i}\right)\;-\;\min\left(r_{i}\right)}$$

where $R_{i}$ is the normalized value of a resource, *“i”*, (e.g., residual energy, available storage, computational capability ), *x* and *y* represents the new range of values (e.g., [0 1]), $r_{i}$ is the current value of the resource *“i”*, $max(r_{i})$ and $min(r_{i})$ is the maximum and minimum values of a resource *“i”* respectively.

After that, the normalized values of resource, *“i”*, are plugged into CH waiting time *“T”* which is a sort of upper layer back-off time and is defined as follows.

(2) $$\begin{array}{cc} T\;=\;\;\frac{1}{\alpha E_{res}+\;\beta C+\gamma S_{av}} & \mathrm{where}\alpha +\beta +\gamma =1 \end{array}$$

where $E_{res}$, *C*, and $S_{av}$ are the normalized values of residual energy, computational capability, and available storage respectively. α, β, and γ are the weights assigned to each factor e.g., residual energy, computational capability, and available storage respectively. It is to be noted that the resource criteria of weights assignment are application specific.

Since the waiting time, *“T”*, is defined based on residual energy, computational capability, and storage capacity of a node, a node with high resources has a low value of *“T”* compared to the nodes with low resources. At the network initialization stage, nodes (high resources) which are capable to become a CH calculate their waiting time *“T”* by employing Equation (2) and wait until that time is expired. If the waiting node(s) receive a CH-Announcement message from any other node during their waiting period, the node(s) stop its timer and associate with the CH. However, if a node does not receive any CH-Announcement message during its waiting time and the waiting time of the node expires, that means there is no other node in its vicinity to become a CH, the node declares itself as CH and forwards CH-Announcement message to the child nodes. Once the CH node(s) is declared, it performs its duties as CH until the available resources (e.g., residual energy) of CH falls below a certain threshold level. The reason is to avoid frequent re-clustering, since frequent re-clustering may degrade the performance of the network. Therefore, when the available resources of current CH falls below a certain threshold level, at that time re-clustering process triggers. At this stage, the other potential nodes (i.e., high resources nodes) except the current CH participates in the CH declaration process. The detailed mechanism of CH declaration phase is provided in Algorithm 1.
**Algorithm 1** CH Declaration Algorithm1:**procedure**CH Declaration2: 3:    resEn←residualenergy4: 5:    avSt←availablestorage6: 7:    CC←computatioanlcapability8: 9:    minEn←min.residualenergy10: 11:    minST←min.storage12: 13:    minCC←min.computatioanlcapability14: 15:    *T*←timerofnode16: 17:    **if** ((resEn>minEn) && (avSt>minST) && (CC>minCC )) **then**18: 19:        NormalizeresEn;20: 21:        NormalizeavSt;22: 23:        NormalizeCC;24: 25:        Assignweightstonormalizedresources26: 27:        calculatewaitingtimeT28: 29:        **if** (*T*≠ 0 && CHmsgRecieved ) **then**30: 31:           Stoptimer;32: 33:        **end if**34: 35:        **if** ((*T* == 0) && (! CHmsgRecieved )) **then**36: 37:           NodedeclareditselfasCH;38: 39:           BroadcastCH-Announcementmsg;40: 41:        **end if**42: 43:    **end if**44: 45:**end procedure**

### 4.2. New Node-Association

Once the CHs are declared, they forward the CH-Announcement messages in their vicinity. The CH-Announcement message is comprised of all the relevant information such as residual energy, computation capability, traffic load, and distance from CH to the sink node.

A child node may receive multiple CH-Announcement messages from multiple CH nodes in the range. Therefore, child node associate itself with the CH which has high residual energy, high computational capability, low traffic load, less distance between CH and sink node and less distance between itself and CH. The rationale behind the consideration of such a large number of parameters for CH selection is to enhance the lifetime of CH in case of application scenarios which generates a massive volume of data. Besides, the CHs are not frequently changed which reduces the control overhead that occurs because of the frequent re-clustering process.

**Figure 2:**
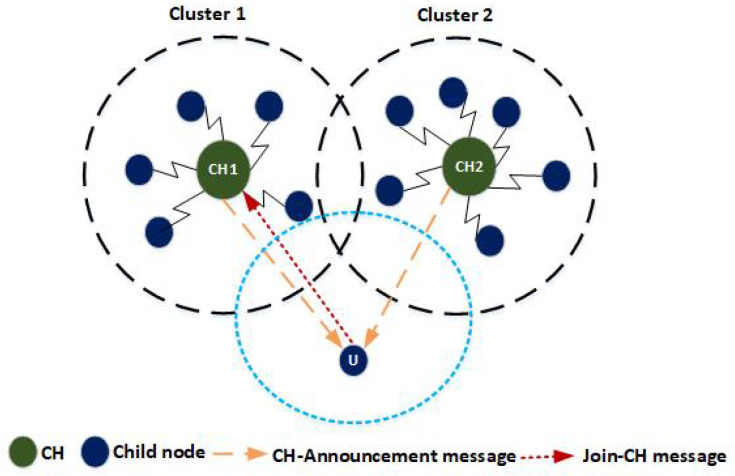
Nodes association process.

Figure 2 illustrates the node association process where the node “U” receives CH-Announcement message from two CHs (i.e., CH1 and CH2). Node “U” has to decide its association with the best CH among CH1 and CH2. Based on the available resources of CH1 and CH2, the node “U” executes new Node-Association process and decides the best CH to join. Let us suppose the CH1 has higher amount of resources than other. CH1 is considered as the best CH for the node “U” to associate with. Therefore, the node “U” sends a Node-Association message to CH1 and associates itself with it.

Initially on receiving CH-Announcement messages from several CHs, the child node(s) calculates Euclidean distance from CH which is defined as follows:

(3) $$d\left(n,CH\right)\;\;=\;\;\;\sqrt{{\left(x-a\right)}^{2}+{\left(y-b\right)}^{2}}$$

where d(n,CH) is the distance between CH and a child node (n), *x*, and *y* are the location coordinates of the child node (*n*) and *a*, and *b* are the location coordinates of CH.

After calculating distance, the child node selects the preeminent CH based on a multi-criteria decision-making technique known as TOPSIS [34,35,36]. The rationale of using a multi-criteria decision-making process is that it allows the node to select the best CH among several potential CHs in its range based on non-homogeneous parameters and the step-wise detailed description of the process by adopting TOPSIS is expressed as follows.

1. Decision Matrix Development
  Consider a child node having *“m”* CHs in its range. The child node organizes the CHs attributes in a decision matrix (*X*) as defined in Equation (4).
  (4) $$X=\;\;\left(\begin{array}{ccccc} X_{\left(1,1\right)} & X_{\left(1,2\right)} & X_{\left(1,3\right)} & . & X_{\left(1,n\right)}\\ X_{\left(2,1\right)} & X_{\left(2,2\right)} & X_{\left(2,3\right)} & . & X_{\left(2,n\right)}\\ . & . & . & . & .\\ . & . & . & . & .\\ . & . & . & . & .\\ X_{\left(m,1\right)} & X_{\left(m,2\right)} & X_{\left(m,3\right)} & . & X_{\left(m,n\right)} \end{array}\right)$$
  where $X_{m,n}$ is the value of *n*th resource criteria of *m*th CH, *m* represents the total number of CHs, and *n* is the total number of the resource criteria such as residual energy, computation capability, traffic load, distance from CH to the sink node, and distance from the child node itself to the CH.
2. Resource Criteria Normalization
  The values of all these criteria do not lie in the same range (e.g., the value of residual energy is not equal to available storage), therefore, the resource criteria must have to be normalized to a common range in order to fairly select the CH. The normalized form of the decision matrix is obtained by employing Equation (5) and is defined as follows:
  (5) $${X^{*}}_{\left(i,j\right)}=\frac{X_{\left(i,j\right)}}{\sqrt{\sum_{j=1}^{n}X{}_{\left(i,j\right)}^{2}}}where\;\;i=1\ldots m,j=1\ldots .n$$
  (6) $$X^{*}=\left(\begin{array}{cccccc} {X^{*}}_{\left(1,1\right)} & {X^{*}}_{\left(1,2\right)} & {X^{*}}_{\left(1,3\right)} & . & . & {X^{*}}_{\left(1,n\right)}\\ {X^{*}}_{\left(2,1\right)} & {X^{*}}_{\left(2,2\right)} & {X^{*}}_{\left(2,3\right)} & . & . & {X^{*}}_{\left(2,n\right)}\\ . & . & . & . & . & .\\ . & . & . & . & . & .\\ . & . & . & . & . & .\\ {X^{*}}_{\left(m,1\right)} & {a^{*}}_{\left(m,2\right)} & {X^{*}}_{\left(m,3\right)} & . & . & {X^{*}}_{\left(m,n\right)} \end{array}\right)$$
  The normalized decision matrix, $X^{*}$ is obtained using Equation (6).
3. Weights Assignment
  After normalization, weights are assigned to each criterion as shown in Equation (7). The $w_{j}^{*}$ in Equation (7) is a weight value allocated to each resource criterion, *j*. The assignments of weights are application-specific (e.g., weights may vary from application to application).
  (7) $$X^{\prime }=\;\;\left(\begin{array}{cccccc} w_{1}^{*}\times {X^{*}}_{\left(1,1\right)} & w_{2}^{*}\times {X^{*}}_{\left(1,2\right)} & w_{3}^{*}\times {X^{*}}_{\left(1,3\right)} & . & . & w_{m}^{*}\times {X^{*}}_{\left(1,n\right)}\\ w_{1}^{*}\times {X^{*}}_{\left(2,1\right)} & w_{2}^{*}\times {X^{*}}_{\left(2,2\right)} & w_{3}^{*}\times {X^{*}}_{\left(2,3\right)} & . & . & w_{m}^{*}\times {X^{*}}_{\left(2,n\right)}\\ . & . & . & . & . & .\\ . & . & . & . & . & .\\ . & . & . & . & . & .\\ w_{1}^{*}\times {X^{*}}_{\left(m,1\right)} & w_{2}^{*}\times {X^{*}}_{\left(m,2\right)} & w_{3}^{*}\times {X^{*}}_{\left(m,3\right)} & . & . & w_{m}^{*}\times {X^{*}}_{\left(m,n\right)} \end{array}\right)$$
4. Ideal Positive Solutions ($IPS^{+}$) and Ideal Negative Solutions ($INS^{-}$)
  *Ideal Positive Solution ($IPS^{+}$)*: The resource criteria where high attribute values such as residual energy, computational capability, and storage capacity are desired are named as $IPS^{+}$. The residual energy of the node is very important, as the overall lifetime of the node depends on its residual energy. Therefore, the highest value of energy is taken as $IPS^{+}$. Similarly, the high storage capacity is also an important criterion, since providing the large storage to a CH prevents congestion and packet drop on a CH. Likewise, the high computational capability reduces the processing delays. All these aforementioned criteria optimize the packet drop rate and enhance the performance of the network.$IPS^{+}$ is computed using Equation (8) and is defined as follows:
  (8) $$IPS^{{}^{+}}=\max\left\{\left(X_{1,j}^{{}^{\prime }}\right),\;\;\left(X_{2,j}^{{}^{\prime }}\right)\ldots \left(X_{m,j}^{{}^{\prime }}\right)\right\},\forall \;j\in \;n$$
  Ideal Negative Solution $INS^{-}$: The resource criteria where the low attribute values such as traffic load on CH, distance from CH to the sink node, and distance from a child node to the CH are desired considered as $INS^{-}$. For instance, if there is a high traffic load on CH, the CH cannot accommodate more data packets from its child nodes due to storage limitations. Similarly, If the distance between the sink node and CH is high, the CH consumes high amount of energy in data transmission towards the sink node. Likewise, if the distance between the child node and a CH is high, the child node has to consume high mount of energy to transmit data. The high values of the aforementioned resource criteria(s) are not beneficial; therefore, these criteria are considered as $INS^{-}$. $INS^{-}$ is computed using Equation (9) and is defined as follows.
  (9) $$INS^{-}=\min\left\{\left(X_{1,j}^{{}^{\prime }}\right),\;\;\left(X_{2,j}^{{}^{\prime }}\right)\ldots \left(X_{m,j}^{{}^{\prime }}\right)\right\},\forall \;j\in \;n$$
5. Difference of each CH from $IPS^{+}$ and $INS^{-}$
  For each criterion, the difference of each resource criteria from the $IPS^{+}$ and $INS^{-}$ are calculated using Equations (10) and (11), respectively and are defined as follows.
  (10) $$\begin{array}{c} \begin{array}{c} X_{i}^{+}=\sqrt{\sum_{j=1}^{n}(X_{\left(i,j\right)}^{{}^{\prime }}\;-\;\;IPS^{{}^{+}}{)}^{2}}\;where\;\;i=\;1\ldots m \end{array} \end{array}$$
  (11) $$\begin{array}{c} X_{i}^{-}=\sqrt{\sum_{j=1}^{n}(X_{\left(i,j\right)}^{{}^{\prime }}\;-\;\;INS^{-}{)}^{2}}\;where\;\;i=\;1\ldots m \end{array}$$
  where, $X_{i}^{+}$ and $X_{i}^{-}$ represent the difference of CH candidate from $IPS^{+}$ and $INS^{-}$ respectively.
6. Ranking Index for final decision
  Equations (10) and (11) compute the difference of each CH node from $IPS^{+}$ and $INS^{-}$ respectively. By doing so, the child node obtains a deviated value of all CHs from $IPS^{+}$ and $INS^{-}$. Since the CHs are heterogeneous in terms available resource, it is highly likely that the deviated value of each CH varies from each other. Based on these deviated values, the child node computes the rank index ($R_{i}$) of each CH node by employing Equation (12). The $R_{i}$ computed from Equation (12) guarantees that the CH with the best available resources is assigned a highest rank as compare to the other potential CHs. Once the child node has the list of CHs with their ranked value, the child node selects the CH which have highest rank. $R_{i}$ of each CH node is computed using Equation (12) and is defined as follows.
  (12) $$R_{i}=\frac{X_{i}^{-}}{X_{i}^{-}\;+\;X_{i}^{+}}\;\;\;where\;\;i=\;1\ldots m$$
  where, $R_{i}$ is the ranking index of *i*th CH candidate node.

The detailed mechanism of node(s) association phase is depicted in Algorithm 2.
**Algorithm 2** New Node(s) Association Algorithm1:**procedure**Node Association2: 3:    CHlist←listofCHsinrangeofnode4: 5:    *i*←*i*th CHs6: 7:    *j*←*j*th resource8: 9:    *w*←weightofresource10: 11:    IPS←IdealPositiveSolution12: 13:    INS←IdealNegativeSolution14: 15:    $R_{i}$←$Rank\;of\;CH_{i}$16: 17:    $a_{i,j}$←*i*th CH,jth critarea18: 19:    **if** (CHlist.length==1) **then**20: 21:        Sendjoin-CHmessage.22: 23:    **end if**24: 25:    **if** (CHlist.length>1) **then**26: 27:        **for** i←1 to sizeOfCHlist **do**28: 29:           GetCHcandidatefromCHlist30: 31:           **for** j←1 to CH−resources **do**32: 33:               InsertCH-resourcesindecisionmatrix34: 35:               Normalizedecisionmatrixe.g.,36:$${X^{*}}_{\left(i,j\right)}=\frac{X_{\left(i,j\right)}}{\sqrt{\sum_{j=1}^{n}X{}_{\left(i,j\right)}^{2}}}where\;\;i=1\ldots m,j=1\ldots n$$37:Assignweightstoeachcriterion38: 39:Constructaweighteddecisionmatrix.40: 41:Compute*IPS^+^*and*INS^−^*42:$$IPS^{{}^{+}}=\max\left\{\left(X_{1,j}^{{}^{\prime }}\right),\;\;\left(X_{2,j}^{{}^{\prime }}\right)\ldots \left(X_{m,j}^{{}^{\prime }}\right)\right\},\forall \;j\in \;n$$$$INS^{-}=\min\left\{\left(X_{1,j}^{{}^{\prime }}\right),\;\;\left(X_{2,j}^{{}^{\prime }}\right)\ldots \left(X_{m,j}^{{}^{\prime }}\right)\right\},\forall \;j\in \;n$$43:Computedistancefrom*IPS^+^*i.e.,$X_{i}^{+}=\sqrt{\sum_{j=1}^{n}(X_{\left(i,j\right)}^{{}^{\prime }}\;-\;\;IPS^{{}^{+}}{)}^{2}}\;where\;\;i=\;1\ldots m$44: 45:Computedistancefrom*INS^−^*i.e.,$X_{i}^{-}=\sqrt{\sum_{j=1}^{n}(X_{\left(i,j\right)}^{{}^{\prime }}\;-\;\;INS^{-}{)}^{2}}\;where\;\;i=\;1\ldots m$46:Calculate*Ri*ofeachCHe.g.,47:$$R_{i}=\frac{X_{i}^{-}}{X_{i}^{-}\;+\;X_{i}^{+}}\;\;\;where\;\;i=\;1\ldots m$$48: 49:SelectCHhavinghighestRankvalue.50: 51:Sendjoin-CHmessage.52: 53:           **end for**54: 55:        **end for**56: 57:    **end if**58: 59:**end procedure**60: 

### 4.3. CH-Acquaintanceship

We present a robust mechanism named CH-acquaintanceship aiming to extend the lifetime of a CH and minimizes packet drop rate in the case of heavy traffic load conditions. To balance the load of CH, a CH may request other CHs having high resources (e.g., residual energy, storage, and computational capability) in its range to balance the load on CH. The overloaded CH sends some of its data to another CH having less traffic load to perform computations on its behalf. By doing so, the CH consumes less energy which in return effectively increases the lifetime of a CH. The overall process of CH-acquaintanceship is explained as follows.

**Figure 3:**
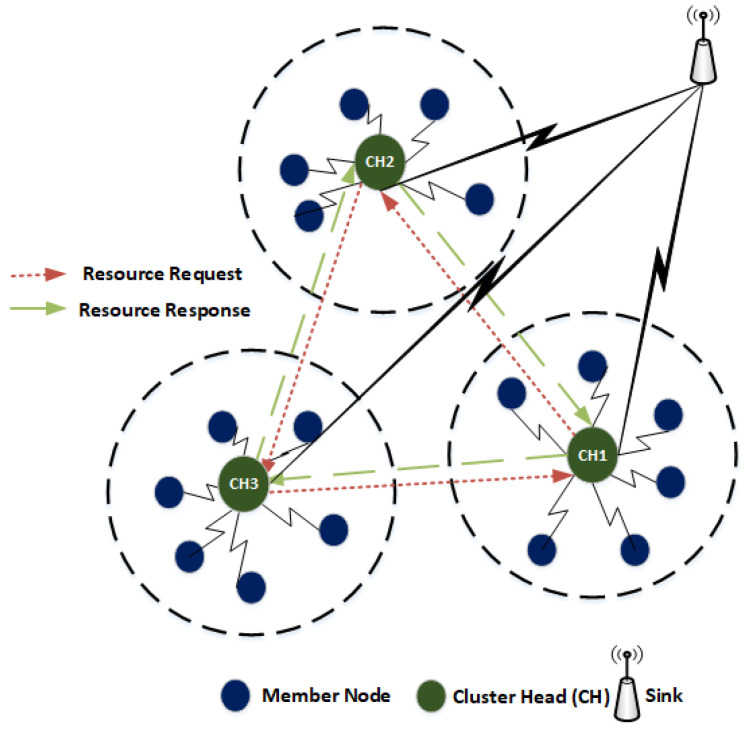
CH-acquaintanceship.

Let us assume a WSN scenario comprises of various type of heterogeneous nodes such as multimedia (audio, video) and scalar (temperature, humidity) as shown in Figure 3. In each cluster, the deployed sensors sense static events (e.g., temperature, pressure, and humidity) and dynamic events (e.g., intrusion, movement of object etc.). Considering two clusters e.g., CH1 and CH2 (Figure 3).

We assume that CH1 has high computational capability and low storage whereas CH2 has high storage and low computational capability. It is assumed that CH1 has a greater number of multimedia nodes as compared to scalar nodes in its cluster. Whereas CH2 has a smaller number of multimedia nodes and more scalar nodes. Moreover, the CH1 is located in the area of high alert and has more chances of intrusion detection as compared to CH2. When an event occurs in the range of CH1, the child nodes of CH1 generates a burst of traffic and forward it towards the CH1. Due to low storage at the CH1, the CH1 may not accommodate the incoming traffic from its child nodes and packets drop may occur. As a result, packets are re-transmitted, thereby, create congestion and collisions in the network. The lifetime of CH decreases as it is highly overloaded due to heavy traffic rate. In order to balance the CH load and to increase the performance of the network in terms of CH lifetime, the CH-acquaintanceship feature allows other CHs to collaborate by sharing their resources to achieve the common goal of a network which is energy optimization. Utilizing the CH-acquaintanceship, the CH1 forwards a certain proportion of its stored data to CH2 and vacates its space for incoming packets from cluster members to avoid packets drop. This reduces the re-transmissions and hence extends the lifetime of a CH. In return, CH2 may use the computational capability of CH1 in worst traffic scenario to optimize its energy consumption and delay.

It is to be noted that the CH-acquaintanceship is strongly dependent on mutual benefits where CHs share resource in return of getting some resources. However, there may be cases where a CH may not have resources to share with other CHs. In such cases, a CH cannot take advantages of CH-acquaintanceship mechanism and may result in degrading the performance of the network. To overcome this issue, we introduced another mechanism named as CH-friendship and is discussed as follows.

### 4.4. CH-Friendship

In cases of bursty traffic, the energy consumption increases due to high processing and transmissions in the network. As a result, the CH resources may exhaust and the CH may not be able receive the data nor it can forward data due to low resources. Therefore, to overcome this situation, we present a mechanism of CH-friendship which balances the load and increases the lifetime of CHs with low resources compared to other CHs. A CH can stay in a network for a longer time, using a friendship mechanism with another CH which have high resources, known as the friend CH. In this mechanism, friendship is established between high resources CH and low resources CH to bring stability in the network. The low resources CH initiates the friendship request as soon as it is overloaded by heavy traffic. The whole operation of CH-friendship is as follows.

Consider a situation where a CH exhaust its available resources due to heavy traffic load, high processing or energy consumption. To prolong the lifetime of CH, a CH initiates the friendship request. The friendship request specifies that the CH has run out of its available resources and requires some resourceful CH to perform some tasks on behalf of a CH having low resources. On receiving friendship request, the CH nodes checks whether they have enough resources to become friends and fulfill the request. The CH nodes which have the capability to fulfill the request may respond with a message for resources availability. After sending the response message, the resourceful CHs wait for response from low resources CH. The low resources CH node selects the potential friend CH based on its available resources (residual energy, computational capability and storage). Upon successful friendship, the low resource CH sends the data to the resourceful node for further processing or storage.

**Figure 4:**
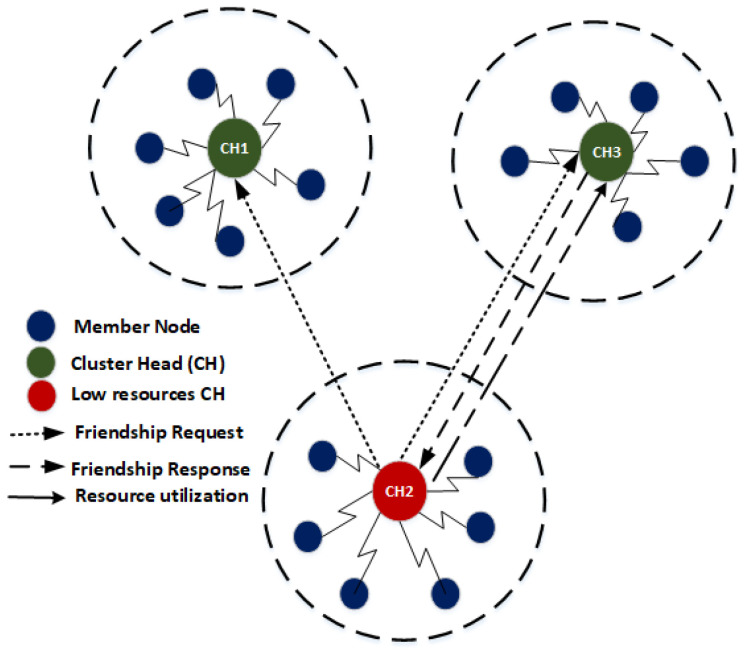
Cluster head (CH)-friendship.

Figure 4 illustrates a heterogeneous WSNs where CH2 run out of its available resources. To avoid the data loss and to make the network stable, CH2 broadcasts friendship request in its transmission range. The request specifies the resources required by CH2 to perform its operation. The receiving CHs (e.g., CH1, and CH3) checks the availability of their resources and decide weather they can fulfill the request of CH2. Figure 4 shows that CH1 has high available resources and its sends a response message to CH2. After receiving a response from CH1, CH2 sends its data to CH1 for further processing and transmission. In this way the CH1 may stay longer in the network and can play the role of CH via friendship mechanism.

## 5. Performance Evaluations

### 5.1. Performance Evaluation Metrics

We conducted multiple experiments to analyze the performance of our proposed scheme and the benchmark protocol using the following metrics.

1. **CH Lifetime:** The CH lifetime is defined as the amount of time a node can act as a CH. In other words, it is the time of CH until re-clustering.
2. **Re-clustering Frequency:** Re-clustering is the process of electing new CH to avoid CH communication failure. Re-clustering mechanism is executed when the available resources of CH go down than a certain threshold (e.g., the energy of CH). Re-clustering frequency is defined as the frequency of re-electing the CHs during the entire network lifetime.
3. **Number of Control Packets:** The control packets considered as overheads and are defined as the packets used for route establishment from a source to a destination e.g., CH announcement, Join CH and TDMA etc.
4. **Packet Drop Ratio:** Packet drop ratio is defined as the ratio of the number of packets lost (not received at receiving node e.g., CH or Sink node) to the total number of sent packets.
5. **Total Energy Consumption:** The energy consumption of a node mainly depends on two main factors (e.g., packet processing and transmissions or receptions) [37]. The cumulative energy consumption of a node is presented in Equation (13) and is defined as follows.
  (13) $$E_{node}=E{}_{proc}+E_{rx}+E_{tx}$$
  where $E_{proc}$ is the energy consumed in processing, and $E_{rx}$ and $E_{tx}$ represents the energy consumed for receiving and transmitting a data packet, respectively.
  The total energy consumption of the network is directly proportional to the number of packets transmitted in the network and is defined as follows.
  (14) $$E_{network}=\;\sum_{i=1}^{n}\left(E_{Proc_{i}}+E_{rx_{i}}+E_{tx_{i}}\right)$$
  where “*n*” represents the total number of packets.

### 5.2. Simulation Environment

We comparatively evaluated the performances of our proposed method with SEED protocol [19] and we considered it as a baseline for our evaluation. For simulations, we used Castalia simulator that was based on OMNET++ [38]. Random topology was used with 100 heterogeneous nodes randomly deployed in a 100 × 100 m area. It was assumed that all nodes were heterogeneous in terms of residual energy, storage (e.g., buffer capacity) and computational capability. The child nodes were resource-constrained, whereas the CHs were resourceful nodes in terms of energy source, storage capacity, and computational capability. The CH nodes required high resources in order to perform their operation efficiently. The optimal percentage of CHs was 5% [16,39], We deployed all nodes (resource-constrained and resourceful) in the ratio of 70% and 30%, where 70% of them were resource-constrained nodes (child nodes) and 30% were resourceful nodes (which could become CH). The rationale of choosing the aforementioned scale was that the child nodes sensed a large environment and transferred the sensed data towards their respective CH node(s). Therefore, the proportion of child nodes was high as compared to CH nodes. The initial energy of high resources nodes varied from 6–10 J and the per bit energy consumption was 0.5 μJ. The reason is, usually the work load on CHs (i.e., aggregation operations, cluster management and collective data transmission) was an order of magnitude larger as compare to work-load on child nodes which only perform sensing operations. Furthermore, the maximum storage capacity of CH nodes was $2^{24}$ bits. Since the proposed scheme considered the packet size of 4000 bits, the maximum number of packets that a node could accommodate in its memory was approximately 4100 packets. All the nodes were location aware (e.g., nodes are equipped with global positioning system (GPS)). The simulation time was 2000 s. Each experiment was conducted five times, and the results were acquired from the average values of measurements. The main simulation parameters are summarized in Table 1.

### 5.3. Results and Discussions

#### 5.3.1. CH Lifetime

**Figure 5:**
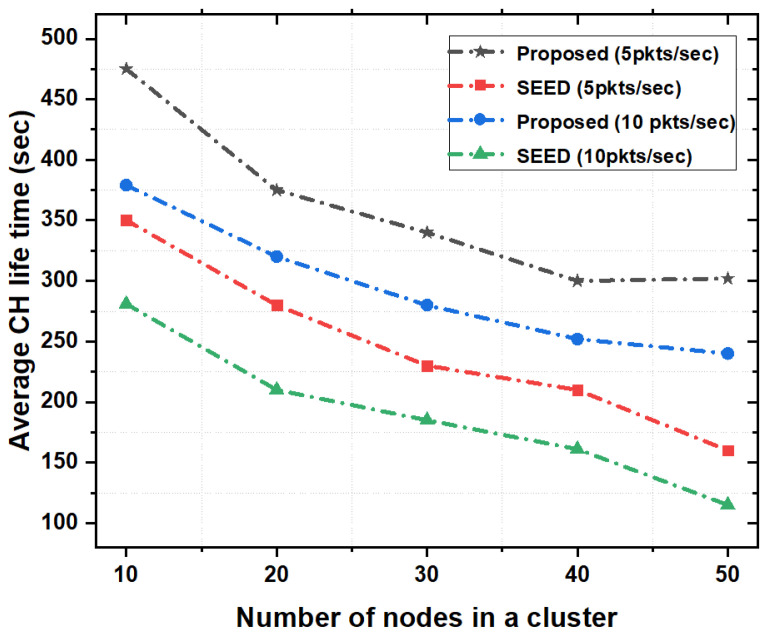
Average CH lifetime as a function of the number of nodes in a cluster.

Figure 5 shows the CH lifetime as a function of the number of nodes in a cluster. The performance of the proposed scheme was analyzed on different cluster sizes (e.g., on average 10, 20, 30, 40 and 50 nodes per cluster) and with two different packet rates (e.g., 5 and 10 pkts/s). As shown in Figure 5, the proposed scheme outperformed SEED protocol in terms of average CH lifetime. It is evident that in SEED protocol, re-clustering happened frequently. The reason is that the SEED protocol only considered the residual energy in the CH declaration process and did not consider other available resources such as storage capacity, and computational capability. Therefore, with increase in nodes density and packet rate, the CH-lifetime decreased. However, the proposed scheme took into account the available resources such as storage capacity and computational capability in addition to the energy. Due to this reason, when the size of cluster increases, the CH stored more data, reduced packet drop and re-transmissions which increased the lifetime of the CH.

#### 5.3.2. Re-Clustering Frequency

**Figure 6:**
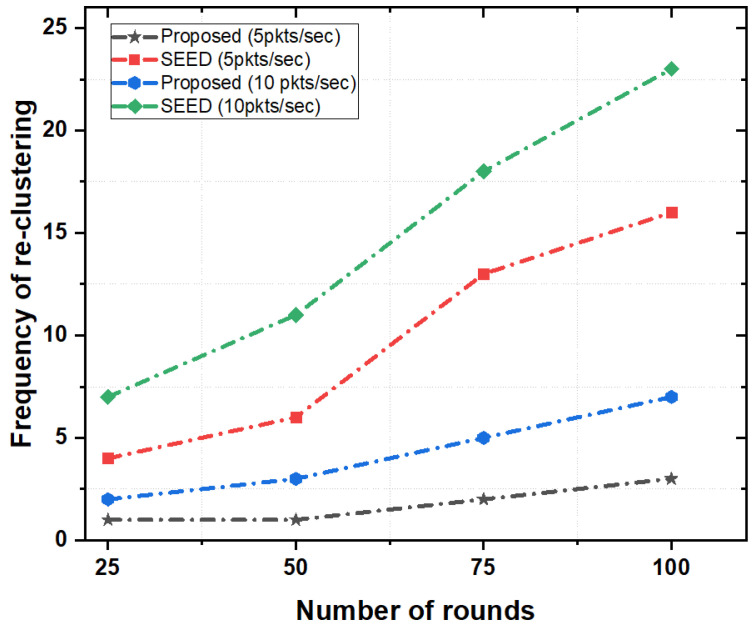
Frequency of re-clustering as a function of the number of rounds.

Figure 6 depicts the frequencies of re-clustering in both the proposed scheme and SEED protocol as a function of the number of rounds (a round is the interval of time during which network topology was created and packets exchanged between source (child nodes), destination (e.g., CH(s), and the final destination (e.g., sink node) takes place). If the frequency of re-clustering was high, control overhead in the network increased. As a result, the overall energy consumption of nodes increased. Figure 6 illustrates that there was less frequent re-clustering in the proposed scheme as compared to the SEED protocol.

In the proposed scheme, the frequency of re-clustering was comparatively low up to 50 rounds and increased slowly in the next 50 rounds for both packet rates (e.g., 5 pkts/s and 10 pkts/s). However, in SEED, the number of re-clustering linearly increased, which meant that the CHs changed more frequently than that in the proposed scheme. The reason is that the proposed scheme reduced the frequent re-clustering by employing a certain threshold value for energy. The re-clustering triggered only when the value of energy fell below a certain threshold of residual energy. However, in SEED, re-clustering happened very frequently without taking into account the status of residual energy. As a result, SEED generated additional control overheads in the network and increased energy consumption.

#### 5.3.3. Number of Control Packets

**Figure 7:**
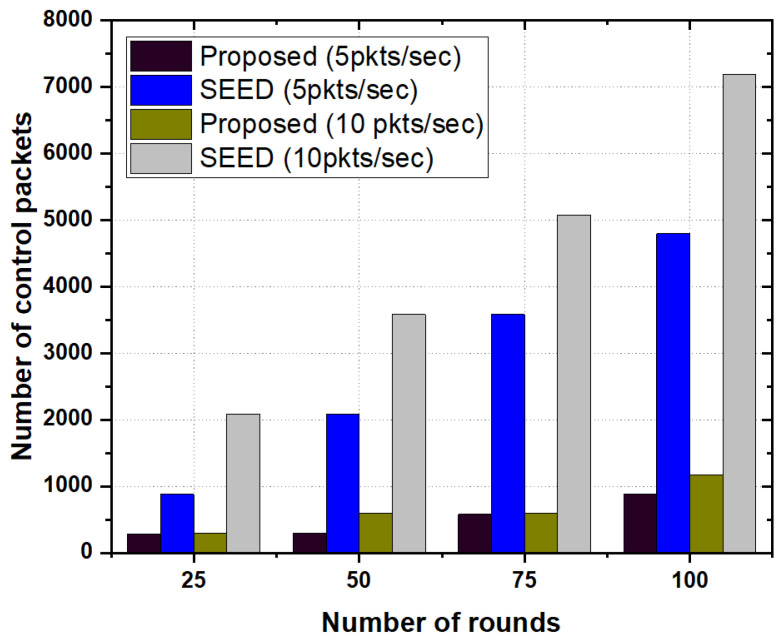
Number of control packets as a function of the number of rounds.

Figure 7 illustrates the numbers of control packets generated by the proposed scheme as well as SEED protocol as a function of the number of rounds.It is evident from Figure 7 that control overhead in the SEED was high as compared to the proposed scheme. We analyzed the behavior of control overhead in the proposed scheme and SEED with varying packet rates e.g., 5 and 10 packets per second. In both cases, the proposed scheme outperformed the SEED. The fundamental reason is that the proposed scheme decreased the frequency of re-clustering, thereby, the number of control packets was significantly reduced in the proposed scheme. On the other hand, in the SEED protocol, since the re-clustering triggered very frequently, the number of control packets increased more than that in the proposed scheme.

#### 5.3.4. Packet Drop Ratio

**Figure 8:**
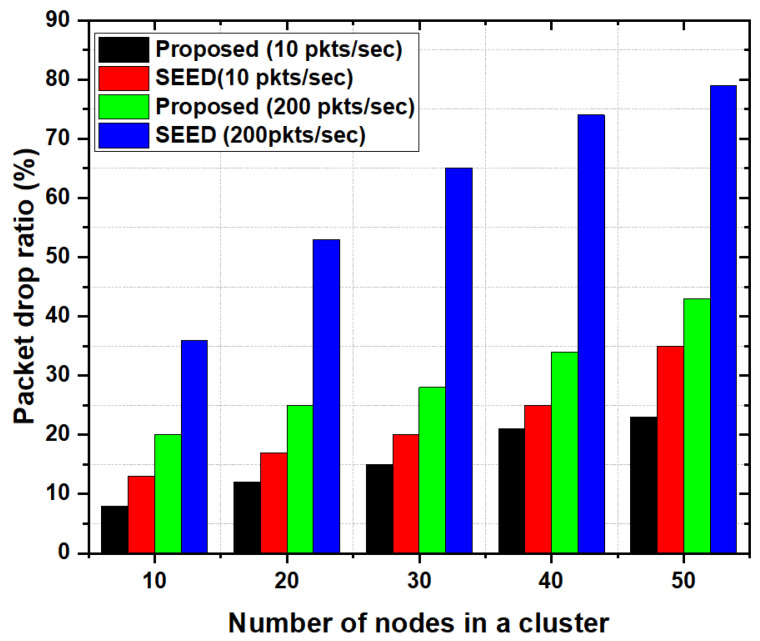
Packet drop ratio as a function of the number of nodes in a cluster.

Figure 8 shows the average packet drop ratio as a function of the number of nodes in a cluster. In the proposed scheme, the packet drop ratio was low as compared to the SEED protocol.

Results show that in SEED, packet drop ratio increased with increase in the number of nodes in the cluster. The main reason is the selection criteria of a CH. In case of SEED, the CH was selected based on residual energy only without considering the storage capacity and computational capability of a CH. Therefore, when the buffer of CH became full, the packets drop ratio increased. In this case, more packets were dropped even though the CH had more energy. However, the proposed scheme considered not only energy but the storage capacity as well. Therefore, more packets were accommodated at CH and the chances of buffer overflow decreased. Furthermore, the proposed scheme also considered the computational capability of CH. Meaning that packets were processed quickly which as a result decreased the packet drop ratio. Due to the consideration of computational capability in the CH selection process, the chances of buffer overflow due to high incoming packet rate decreased significantly. Consideration of these two parameters in addition to energy (e.g., storage and computational capability) increased the chances of CH to accommodate more packets which in turn reduced the packet drop ratio.

Furthermore, situation(s) may arise where buffer overflow occur due to huge traffic volume. Despite the fact that the proposed scheme considered computational capability and storage capacity, it may fall short for heavy traffic scenarios, meaning that in heavy traffic conditions, CH may not accommodate incoming traffic and may result in packets drop. To overcome this situation, the proposed scheme utilized the additional features e.g., CH-acquaintanceship and CH-friendship. We evaluated the effect of CH-acquaintanceship and CH-Friendship features for heavy traffic scenarios by increasing the packet rate in the network. The packet generation rate was increased by 20 times (e.g., on average a node was transmitting 200 packets/s). As shown in Figure 8, the proposed scheme significantly reduced the packet drop ratio in case of heavy traffic conditions. The main reason is that the proposed scheme employed the CH-acquaintanceship and CH-friendship feature which allows the low resources CH to share its load with resourceful CH in its transmission range to reduce the packets drop ratio. Therefore, in the proposed scheme when there was a sudden increase in traffic load on the CH, it started sending the packets towards neighboring CH. By doing so, the CH’s storage got free and there were rare chances of packet drop on that CH. In contrast, in SEED, the packet drop ratio increased abruptly, since no CH-acquaintanceship and CH-Friendship mechanism were employed in this case.

#### 5.3.5. Total Energy Consumption

**Figure 9:**
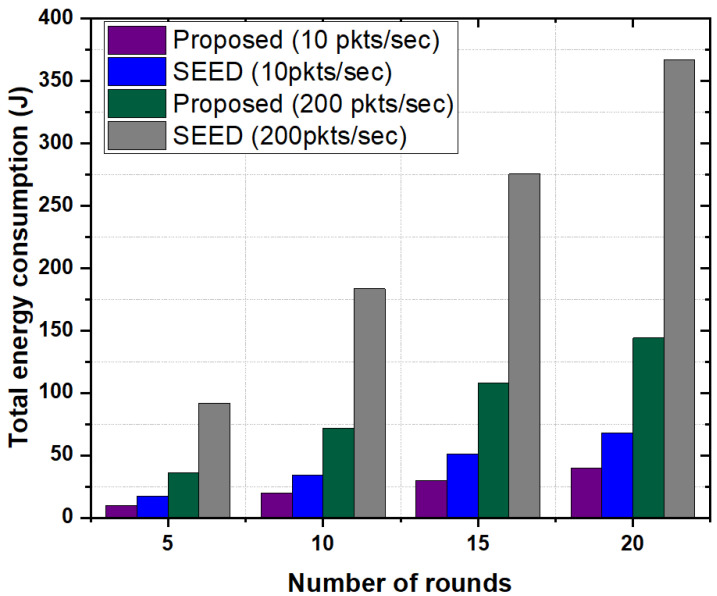
Total energy consumption as a function of the number of rounds.

In this experiment, total energy consumption of network in the proposed scheme and SEED were evaluated against the number of rounds. For normal traffic scenario, packet rate of 10 packets per second was used. Figure 9 depicts that the proposed scheme had lower energy consumption compared to SEED in all rounds. Due to frequent re-clustering in SEED, the control messages were transmitted frequently to rebuild the cluster, which increased energy consumption of the nodes. Another reason is that SEED did not consider the storage capability and computational capability of node in the CH declaration process. Therefore, in high traffic conditions, a CH may not store the incoming data packets which in turn creates buffer overflow. Due to buffer overflow packet drop ratio increases thereby result in high energy consumption. However, the proposed scheme considered the storage capacity and computational capability along with residual energy in the CH declaration process which in turn minimized the re-clustering frequency, meaning that if the residual energy of CH went below a certain threshold, the re-clustering process executed. This significantly reduced the overall energy consumption of nodes.

**Figure 10:**
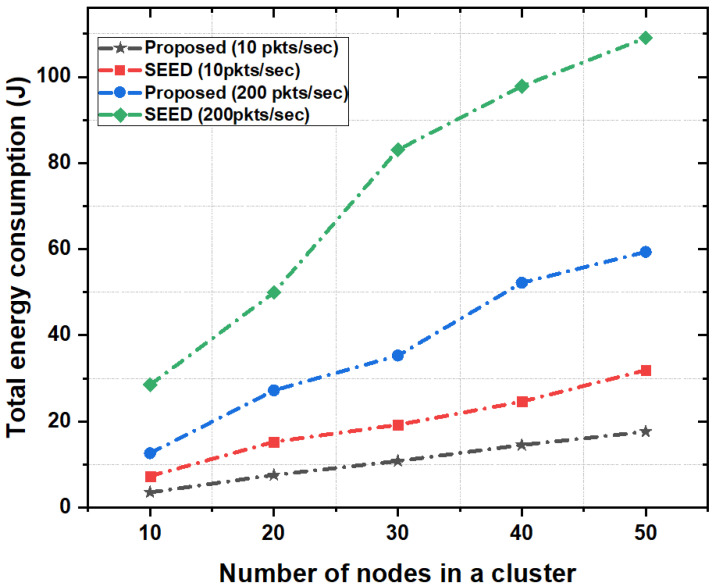
Total energy consumption as a function of the number of nodes in a cluster.

In order to show the effect of bursty traffic on energy consumption, the packet rate of 200 packets per second was used. Results show that the proposed scheme significantly reduced the total energy consumption of network (around 60%) as compared to SEED. The reason is that in the proposed scheme CH shared its load with the neighboring high resource CHs which reduced the packet drop rate and packet re-transmissions. Therefore the overall energy consumption of network reduced. In contrast, SEED did not employ any mechanism such as CH-acquaintanceship and CH-friendship to optimize energy consumption of nodes in bursty traffic scenarios. Moreover, we also analyzed the performance of proposed scheme by varying the number of nodes in a cluster as shown in Figure 10. Results show that the proposed scheme outperformed the SEED protocol in each case and consumed on average of 50% less energy compared to SEED. In the proposed scheme, increase in the number of nodes and packets in a cluster did not have high impact on energy consumption. Since the CH had high storage and computational capability, the CH stored more data from child nodes and reduced the energy consumption due to frequent re-transmissions. Additionally, the CH-acquaintanceship and CH-friendship features of proposed scheme reduced the overall energy consumption.

## 6. Conclusions

In this paper, we proposed an efficient clustering mechanism to enhance the network lifetime and optimize the overall energy consumption of nodes. The proposed scheme provides an efficient CH declaration mechanism to minimize the re-clustering frequency which minimizes the control packets overhead and enhances the CH lifetime. In the nodes association process, the nodes employ a multi-criteria decision-making technique TOPSIS to select the best CH among several potential CHs candidates. Moreover, the scheme also provides mechanisms such as CH-Acquaintanceship and CH-Friendship to reduce the energy consumption, optimize the workload, minimize the packet drop rate and extend the lifetime of CH. Simulation results reveal that the proposed scheme optimizes the energy consumption, extends the network lifetime, minimizes re-clustering frequency and control overhead, and reduces packet loss as compared to the existing clustering scheme. 

## Figures and Tables

**Table 1:** Simulation Parameters.

Parameter	Value
Simulator	Castalia v-3.2
Area	100 × 100
Total number of sensor nodes	100
Node distribution	Random
Initial Energy of nodes	6 J–10 J
MAC	Tunable Mac (T-Mac)
Packet rate	5 pkts/s, 10 pkts/s, 200 pkts/s
Packet Size	4000 bits
Energy Consumption	0.5 μJ/bit
Buffer size	Max $2^{24}$ bits
Propagation Model	Log-Normal Shadowing Model
Simulation time	2000 s
